# Predicting Fatigue in Long Duration Mountain Events with a Single Sensor and Deep Learning Model

**DOI:** 10.3390/s21165442

**Published:** 2021-08-12

**Authors:** Brian Russell, Andrew McDaid, William Toscano, Patria Hume

**Affiliations:** 1 Sports Performance Research Institute, Auckland University of Technology, Auckland 0632, New Zealand; patria.hume@aut.ac.nz; 2 National Aeronautics and Space Administration, Ames Research Center, Moffett Field, CA 94043, USA; William.b.toscano@nasa.gov; 3 Department of Mechanical Engineering, University of Auckland, Auckland 1142, New Zealand; andrew.mcdaid@auckland.ac.nz

**Keywords:** fatigue, cognitive, physical, executive decision-making, psychophysiology, artificial intelligence, deep learning, multi-day missions

## Abstract

Aim: To determine whether an AI model and single sensor measuring acceleration and ECG could model cognitive and physical fatigue for a self-paced trail run. Methods: A field-based protocol of continuous fatigue repeated hourly induced physical (~45 min) and cognitive (~10 min) fatigue on one healthy participant. The physical load was a 3.8 km, 200 m vertical gain, trail run, with acceleration and electrocardiogram (ECG) data collected using a single sensor. Cognitive load was a Multi Attribute Test Battery (MATB) and separate assessment battery included the Finger Tap Test (FTT), Stroop, Trail Making A and B, Spatial Memory, Paced Visual Serial Addition Test (PVSAT), and a vertical jump. A fatigue prediction model was implemented using a Convolutional Neural Network (CNN). Results: When the fatigue test battery results were compared for sensitivity to the protocol load, FTT right hand (R^2^ 0.71) and Jump Height (R^2^ 0.78) were the most sensitive while the other tests were less sensitive (R^2^ values Stroop 0.49, Trail Making A 0.29, Trail Making B 0.05, PVSAT 0.03, spatial memory 0.003). The best prediction results were achieved with a rolling average of 200 predictions (102.4 s), during set activity types, mean absolute error for ‘walk up’ (MAE200 12.5%), and range of absolute error for ‘run down’ (RAE200 16.7%). Conclusions: We were able to measure cognitive and physical fatigue using a single wearable sensor during a practical field protocol, including contextual factors in conjunction with a neural network model. This research has practical application to fatigue research in the field.

## 1. Introduction

### 1.1. Why We Need to Measure Physical and Cognitive Fatigue in the Field

Measures of physical and cognitive fatigue are needed in the field to improve performance and help improve safe participation in outdoor environments.

Physiological and cognitive fatigue in field environments directly affects performance as a person modulates decisions based on contextual input to maintain resources [1]. Various fields where operational safety is related to fatigue have been investigated, including pilots [2,3], motor vehicle drivers [4,5,6,7,8,9], firefighters [10,11], and shift workers [12]. Physical fatigue relates to reduced force, endurance, level of effort, strength, speed, and coordination [13]. Levels of performance may be modulated by physical load, sleep, nutrition, and psychological factors based on mission duration, pain, levels of perceived exertion [14,15,16,17], intensity, and time on task [18]. Hill [19] won the Noble prize for his work on skeletal muscle and maximum oxygen uptake.

The interaction of central fatigue and motivating factors have been modelled in various forms: Borg’s [20] Rating of Perceived Exertion (RPE); Millet’s [21] Flush model for pacing strategies in ultra-marathons; Noakes’s [17] central fatigue model; and Venhorst’s [22] bio-psycho-social model.

Cognitive fatigue can be viewed as a combination of goal, adaption, and reward trade-offs, including the energetic requirements to achieve a goal [23,24]. Performance psychology [25,26] describes performance as recalling one’s knowledge, skills, and abilities during an event. Cognitive and physical fatigue have a complex interaction of over-lapping redundant systems [27].

### 1.2. How We Can Measure Physical and Cognitive Fatigue in the Lab and the Field

Mental and physical fatigue have been researched in the lab using different sensing modalities including computer interaction [28], accelerometery, electroencephalogram (EEG), electrooculography (EOG) [29], electromyography (EMG), and electrocardiograph (ECG) [16,30,31,32], however, these techniques are not always practical in a field setting.

Assessment of performance and fatigue has been studied [3] with multiple sensors and neural networks. However, they have not been validated in the field with noise sources such as terrain, slope, and obstacles. Enoka [33] noted that lab-based experiments such as maximum voluntary contractions (MVC) result in task dependency that do not translate into field performance. The reduction of separate effects does not equate to overall performance. The only way to determine performance reductions from fatigue is to measure the response to loads in the field.

Field applications require the number of sensors to be minimized while performing challenging multiday events and to not distract the operator from their mission tasks or add to logistical loads when deploying technology into an operational environment. Where multiple sensors would aid accuracy and redundancy, they may lead to lack of deployment of the entire system, hence a minimum viable solution to maximize use by operators is desirable. A review of sensors used for measuring occupational fatigue [34] showed that the most effective sensors were heart rate and accelerometry. Smartphones with multi-channel inertial sensors and deep learning models have been used for human activity recognition [35,36] in controlled environments for complex activity types. A review of physical and cognitive fatigue has shown a relationship of heart rate and accelerometry with muscle activity, proprioception, and changes in gait [37,38,39]. Gait has been shown to change physical performance with increased mental fatigue [9,16,40], goals [41], and reduced executive function [42]. Terrain has been shown to influence gait and accelerometry readings [43].

Traditional machine learning with feature extraction has been used in applications such human activity recognition [43,44], however this approach assumes the features of interest are known and calculatable. Deep learning uses models which automatically determine feature morphology and significance in the data which may not be observable with traditional statistics and data analysis. Deep learning has been used for areas such as wakefulness detection with accelerometry and ECG [45] and fatigue estimation by Gordienko et al. [46] showed positive results with a repetitive exercises in the gym. Recurrent neural network (RNN) and long short-term memory (LSTM) are often cited as the preferred models for time series data [47]. Convolutional neural networks (CNN) have also been used for time series data [43,48] and do not suffer from the stability issues of RNNs while enabling parallel processing which is not possible with RNN type models. CNN models have shown good performance on physiological time series data for emotion classification, [49] and mental fatigue [50] using EOG, which is not generally practical in field operations with high levels of activity. Accelerometry has been shown to be affected by cognitive fatigue [51].

The aim of this study was to:

- Determine whether cognitive and physical fatigue could be accurately predicted by an AI model using data from a single sensor capable of being worn in an endurance activity for multiple days, measuring acceleration and ECG in an outdoor environment with voluntary activity.
- Additionally propose a protocol for data collection in an unsupervised remote environment with no manual labelling by the participant
- Determine if environmental parameters would affect accuracy, including; random activity, self-pacing, terrain surface (concrete, gravel, dirt, mud grass), and slope (flat, up and down slopes)

## 2. Materials and Methods

### 2.1. Ethics

The researcher’s university ethics committee (AUTEC 18/412) approved all procedures in the study and the participant gave written informed consent prior to participating in the study.

### 2.2. Protocol—Physical and Cognitive Load and Performance Assessments

A protocol was developed that included self-paced running in an unstructured mountain environment and standard performance assessments with no distractions in a laboratory for comparison.

**Figure 1:**
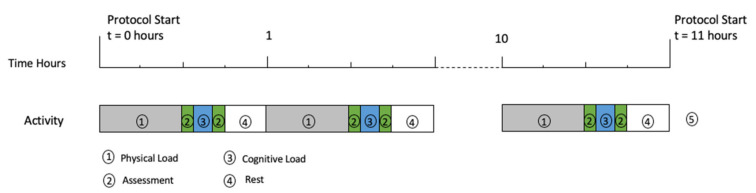
Fatigue Protocol.

The protocol was developed using physical and cognitive loads in excess of a participants’ critical power [52] to induce fatigue. A one-hour period of fixed load was repeated until the participant voluntarily ceased the protocol. No restart was allowed. Physical load was provided by a trail run (3.8 km, 200 m vertical gain), and cognitive load was provided by 10 min Multi Attribute Test Battery (MATB) [53] (Figure 1).

A goal was set as 100 km distance, 5200 m (17,000 feet) total climb, and 26 h’ time in order to address motivation [14] and psychological perception of pain [54]. The course was prescribed to cover various slope angles and terrain types (concrete, gravel, dirt, grass, boulders) and obstacles (trees, river, gate, fence) and to not require active navigation for safety under fatigue and reduced decision-making capacity [55]. Speed was rewarded by earlier completion of the hourly protocol, resulting in a larger rest period per hour.

For clinical comparison, a battery of performance assessments were completed on an iPad Pro (Apple, Cupertino, CA, USA) using a custom application, implementing tests built with an Apple Research Kit [56]. The battery of assessments was chosen because they have previously shown sensitivity to the protocol loads and fatigue-related diseases, Table 1. These included assessments used for fibro myalgia [57], Parkinson’s [58], and physical [16,59] and cognitive fatigue [60]. Assessments used included Stroop, Finger Tap Test, FTT, Trail Making A, Trail Making B, paced serial addition test, PVSAT, memory, and jump height.

### 2.3. Data Preparation

The participant wore a chest-mounted BioHarness (Medtronic, MN, USA) [68,69] for acceleration data (100 Hz, vertical x-axis, sagittal z-axis, lateral y-axis) and electrocardiogram (ECG) (250 Hz) and a Garmin Forerunner GPS (Garmin, KS, USA) wrist watch (1 Hz, horizontal accuracy 6 m) to assist with labelling and location.

**Figure 2:**
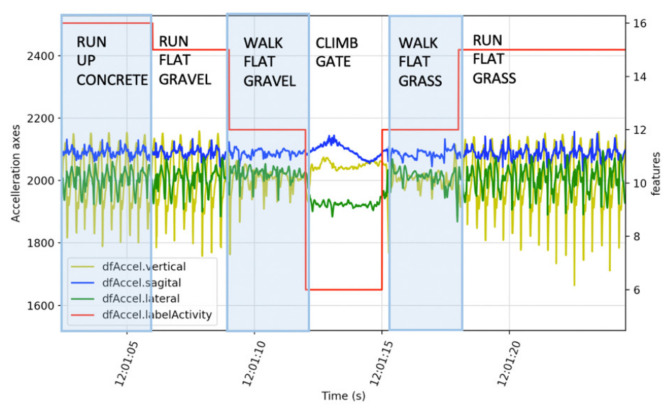
Time series example of the one-off activity “climb gate” verses repetitive data “run” and “walk”.

The trail was divided into twenty-three sections separated by waypoints defined by a change in terrain surface, slope, or obstacle. Terrain descriptors were validated against video (GoPro Hero 4, Garmin, KS, USA). Slope was determined from a mean of GPS altitude measurements at each waypoint. Waypoint location was determined from Google maps to an accuracy of 10 cm. Time at a waypoint was determined when the subject was closest. Walk and Run activity labels were defined by cadence from vertical axis accelerometery zero crossings (100 < Walk < 150 < Run steps per minute) as described in Russel et al. for human activity recognition [43]. Identification of crossing obstacles was based on geographic location and manual observation of the acceleration waveforms Figure 2. Time resolution for labelling was one second.

### 2.4. Convolutional Neural Network

Figure 2 shows the multi-channel 1-D Convolutional Neural Network (CNN) that was selected to allow learning on separate channels and cross correlation into a single regression output value. The training label was FTT up-sampled to 250 Hz. Data were split by activity type and segmented by input window length. The initial model width for all hidden layers was set at 256, which was approximately one second of data. The model implemented the Adam optimizer and mean absolute error (MAE) as the error term during training. Randomized train test split ratio was 0.33.

Hyper parameter tuning, included window size for each activity type, was performed (64, 128, 256, 512). The lowest MAE activity was selected for further model optimization of hidden layer widths. Optimization was performed separately for three datasets: acceleration; ECG; and combined acceleration and ECG. The final model for comparison was selected for lowest MAE. Performance was assessed using the mean absolute difference (MAE_200_), and range of absolute difference (RAE_200_), between the label values and the average of 200 predictions. RAE was of interest as it indicated the largest error possible when the trained model was used to predict a fatigue value in the future.

### 2.5. Statistics

Linear regression (Pearson correlation R^2^) was performed on each performance test to assess sensitivity of the protocol. The performance test results were normalized across the protocol and linearly interpolated to give a long-term linear fit (LTLF). The same tests with highest R^2^ were up-sampled to 250 Hz using inter-test interpolation (ITI), as ITI includes short term fatigue and recovery. LTLF is more representative of long-term fatigue but is only possible with a research protocol designed with a constant load over time. ITI is needed for random field predictions where no assumptions can be made about overall loads.

Time series data were normalized using feature scaling via Equation (1) in preparation for training the CNN. ECG data were base line corrected. All accelerometer axis (x, y, z) and ECG data were transformed into an array (D, W, F) with D rows, W window width, and F number of features.

(1) $$x_{\mathrm{new}}=\frac{x-x_{\min}}{x_{\max}-x_{\min}}$$

## 3. Results

The participant voluntarily ceased the protocol at 11 h (2200 m vertical climb, 41.8 km) due to perceived exhaustion.

**Figure 3:**
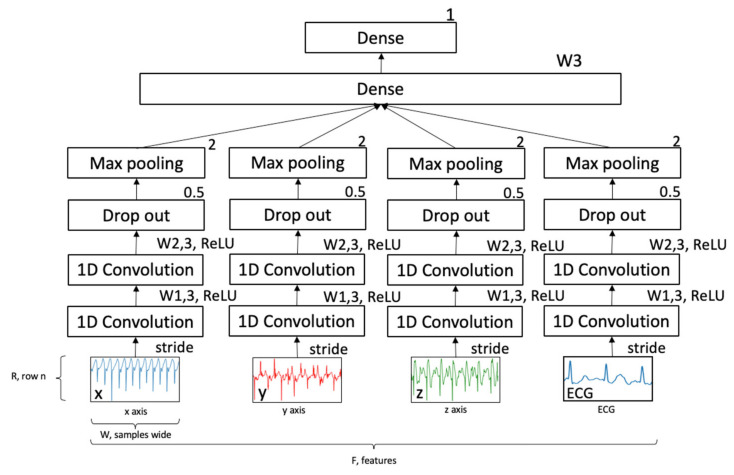
Structure of CNN.

Figure 3 shows the representative input to the CNN of the gait waveforms of vertical acceleration on tarseal and dirt at different fatigue levels. Each plot is 50 steps triggered at zero g and plotted with the median waveform in a thick black line. Inter-step variation in acceleration and morphology can be observed between surfaces (a) tarseal and (b) dirt. The changes in waveform shape between surfaces was likely due to surface hardness and variations in surface texture uniformity. Across the protocol, variation was likely due to fatigue reducing peak forces and subsequent gait adaption, as seen on the plots at point (c).

**Figure 4:**
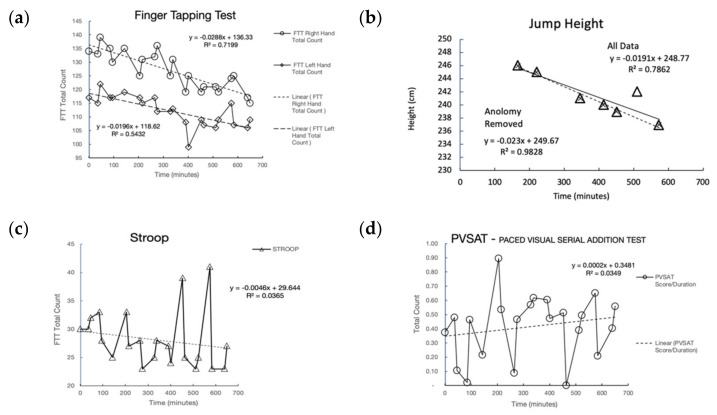
Performance Tests (**a**) FTT, (**b**) Jump Test, (**c**) Stroop, (**d**) PVSAT.

A subset of performance tests (FTT, Jump test, Stroop, PVSAT) completed in the protocol are shown in Figure 4. Jump height and FTT-right-hand were most sensitive to the fatigue protocol.

Figure 4a shows FTT-right-hand and the slower non dominant left hand with separate linear regression lines. Inter-test variation was observed between physical and cognitive tests, with an overall trend having a negative slope showing performance was decreasing over time. Correlation results for all tests are shown in Table 2. Jump height shown in Figure 4b was performed after each physical load period and showed high correlation (R^2^ 0.78) with the protocol. Stroop shown in Figure 4c had two outliers and showed moderate correlation (R^2^ 0.5) with the outliers removed. PVSAT shown in Figure 4d was not correlated with the protocol load. Trail making A (R^2^ 0.29) and spatial memory (R^2^ 0.28) were somewhat correlated to post cognitive load. Trail making B (R^2^ 0.22) was somewhat correlated to post-physical load.

**Figure 5:**
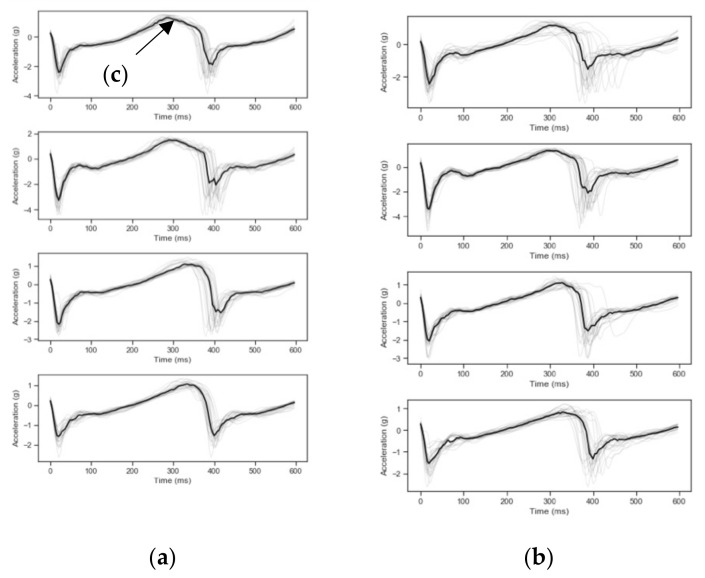
Acceleration for activity “Run Down” over protocol time (0, 3, 8, and 10 h) for surfaces (**a**) tarseal, (**b**) dirt, and (**c**) feature of interest over fatigue.

Figure 5 shows the variation of gait for four periods in the protocol illustrating the variation to the accelerometer waveforms for both fatigue levels and terrain.

**Figure 6:**
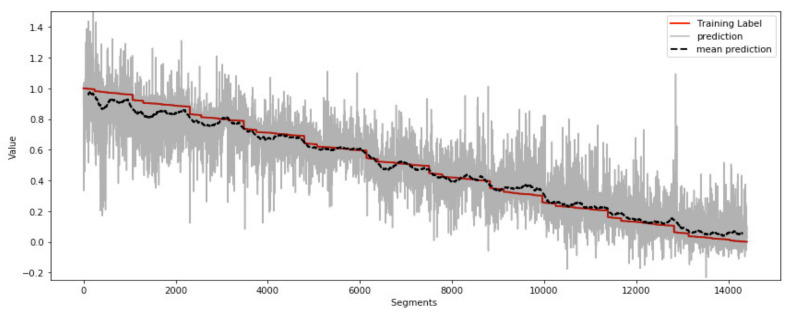
Training Results (BLACK) for CNN with activity ‘run down’ with training label (RED) and individual predictions (GREY).

A training result is shown for a single activity ‘run down’ in Figure 6 for data window 128, epoch 100, individual predictions (light grey), and rolling average of 200 predictions (black). The label for FTT (red) inter-test linear interpolation with discontinuities between time periods due to concatenation.

**Figure 7:**
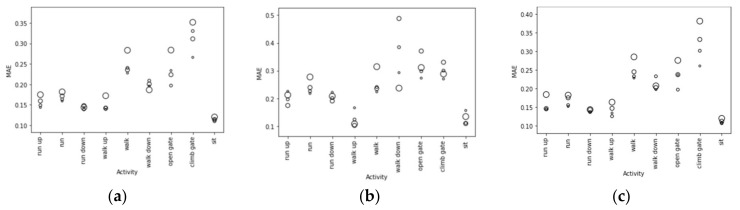
Training Loss, MAE, for (**a**) Accelerometer, (**b**) ECG and (**c**) combined ECG and accelerometer, epoch = 100, circle radius set by data window width (64, 128, 256, 512).

A total of 108 machine learning experiments were performed to test which input data width and activity type gave the best MAE. Initially, a fixed CNN topology was used (Epoch 50, Batch 256, layer 1 filter 256, layer 2 filter 256, dense layer 128, overlap = 0). Three data group results were compared for: acceleration, ECG, and combined acceleration with ECG. These three conditions were tested for each activity type (‘run’, ’walk’, etc.) over four data window widths (64, 128, 256 and 512). The results for these experiments are shown in Figure 7 by activity, where circle diameter is data window width. Minimum MAE was at ’walk up’ (window width 256, MAE 0.105, samples 1,534,500, windows 5994) and ‘sit’ (window width 256, MAE 0.116, samples 2,662,750, windows 10,401). However, sit was not included as it took place in the lab for cognitive testing. Samples were more numerous for ‘run down’ (window width 256, MAE 0.181, samples 1,843,749, windows 7202) and still gave a larger minimum MAE. This indicates that total sample count is not the main influence on MAE, however the activity with considerably lower samples did show larger MAE values, ‘walk down’ (stride 512, MAE 0.309, samples 20,000, windows 78).

Further experiments were performed for acceleration and ECG with ‘walk up’ to optimize the CNN model hyperparameters, various widths of the first two convolutional layers, and the dense layer. The lowest MAE was found to be the following model: Conv1D 128, Conv1D 128, max_pooling, flatten, dense 128, dense 1.

Table 3 shows the total samples per activity and results for MAE and RAE with the training labels using two methods, linear fit, and inter-test interpolation, window width 128, epoch 100, batch size 256, and a rolling window average of 200 predictions. There was no result for activity of ‘walk-down’ as the total samples divided by the window width of 128 was 156, which was less than the rolling average of 200 predictions. Activity ‘Walk Up’ gave the lowest MAE for both linear interpolation and inter-test interpolation of label data. Activity ‘Run Down’ gave the lowest range of errors, indicating it may be a better activity for field prediction.

## 4. Discussion

A protocol for cognitive and physical fatigue was performed in the field, with voluntary activity selection and voluntary pacing over various terrain slopes and surfaces. Jump height and FTT-dominant-hand were most sensitive to the protocol. FTT-non-dominant-hand and Stroop were moderately sensitive. FTT was the most sensitive and biomechanically non-specific, as the legs were exposed to physical load and the arms–hand–fingers were tested for neuromuscular performance. It is likely Stroop would be more sensitive if the protocol included sleep deprivation. Spatial Memory was mildly correlated to the cognitive load.

The experiment showed that a field protocol of cognitive and physical load in excess of a critical power will cause failure and modulate standard objective measures of cognitive and physical performance. Mental and physical fatigue led to earlier-than-anticipated termination of the protocol, which aligned with previous studies [16,40].

The use of a machine learning model was required due to the complex gait waveform morphology variations throughout the protocol. The results for acceleration, ECG, and combined acceleration and ECG are shown in Figure 7 across various stride lengths from 64 to 512 samples. While the activity ‘sit’ had low MAE showing how a controlled environment could give good results, our work aimed to determine if it was possible in an uncontrolled field-based environment. Activity ‘walk up’ had low MAE for both inter-test interpolation and long-term linear fit. ‘Run down’ had the lowest RAE. It is recommended that RAE is used, as this represents the results you would get when using the model in the future for inference.

This experiment showed how a single sensor could be used in conjunction with a CNN model to give accurate results of cognitive and physical fatigue equivalent to gold standard objective tests; FTT and Vertical Jump Test. Best results were obtained when model training was specific to activities such as ‘run down’ and ‘walk up’. MAE and RAE performed well for a rolling window of 200 continuous predictions of 102 s. This intuitively makes sense that any one step in a persons’ gait may be influenced by objects, surface, and other distractions, and it is best to use multiple steps of a persons’ gait to determine a fatigue result. Winter [70] showed that the cadence in steps per minute on a uniform surface varied from 84.7 ± 10.4 for slow to 121.6 ± 5.3 for fast.

The input window size of the CNN model has an optimum size. Too small does not allow a full gait or ECG waveform to be analyzed, and too large significantly reduces the number of training samples.

Tests that had the highest sensitivity to the protocol, and indicated a central fatigue component, were the jump test (high physical load on the legs) and the FTT (utilized hand digits which were not significantly utilized during running). Cognitive tests were less sensitive to the protocol, indicating there may have been a mismatch between cognitive and physical loads.

The effectiveness of the protocol was encouraging as it provided proof of concept for translational research to be undertaken in outdoor environments. Future work could examine how team workload and tactical decision-making can be adjusted for cognitive and physical fatigue in real time with no additional data entry for soldiers on multiday missions. Recovery during training missions could be assessed without researchers being present. Adventure sports people could gain insight into their cognitive and physical fatigue, enabling informed training plans. Work rest cycles could be adjusted, and critical tactical and navigation decisions can be chosen based on periods of highest cognitive performance.

This feasibility study researched approaches of protocol design, error sources, calibration techniques, data collection, validation, labelling, and data processing. Given the lessons learnt, data gathering and processing needs to be more automated to reduce the high processing load that occurred for the one participant in this study. Further work is needed to test inter-subject variability to the protocol, test–retest accuracy of the prediction model, longer duration, and additional fatigue modulators including sleep, pain, discomfort, and nutrition.

### Limitations

Limitations in validating the experimental objective include a linear protocol and the limited amount of comparison tests, however, this is a natural limitation in the field of cognitive assessments in the field. A long-term linear fit was appropriate for this protocol as the repetitive load could be assumed constant over the longer-term time frame. A random field assessment with no defined load protocol would require training using inter-test interpolation to allow for stochastic loads and recovery cycles. A constant long-term load was required to fit a machine learning model. Future work could compare the results in a long-term non periodic protocol. 

The limitations of this test were the duration and the use of a single participant to initially prove the feasibility of the protocol and approach. Further research is required around increasing the duration of the protocol, possibly by reducing the hourly physical load. Additional studies over longer periods are required to generate cognitive fatigue that includes sleep deprivation. The test battery should include assessments immediately after large vertical assents to gather insight into short-term recovery. The addition of cognitive loads and assessment significantly affected the rate of perceived exertion. Future protocols should halve the physical load to lengthen the time to failure. Additionally, this method requires more participants to compare inter-person sensitivity and variability. 

## 5. Conclusions

This paper showed that a single wearable sensor could be used in conjunction with a neural network model to determine cognitive and physical fatigue without performance tests being required during an operation in an outside unstructured environment. This research has the potential to increase safety and operational performance in high-risk environments by indicating the possibility of replacing traditional performance tests with a single wearable device. This work is novel, to the knowledge of the authors, in developing a field-based protocol for human performance with no direct supervision and modulation from ground surface, slope, fatigue, and task motivation. Future research is required for more participants and will require further automation of data labelling to process field data with self-pacing activities.

## Figures and Tables

**Table 1:** Assessment Battery.

Assessment	Bio-Psycho-Central Performance	Reference
Finger Tap Test	Neuro muscular fatigue	[59]
Stroop	Cognitive flexibility and selective attention	[61]
PVSAT	processing speed, attention, working memory	[62]
Trail Making A and B	Motor and executive impairment	[63]
Corsi Block test	Spatial memory, Working memory	[64,65]
Vertical Jump	Neuromuscular fatigue	[66]
Rating of Perceived Exertion	Perceived level of exertion	[16,20,67]

**Table 2:** Performance test sensitivity, R2, to the protocol load.

Test	All Tests	Post Physical Load	Post Cognitive Load
Jump	0.78	-	-
Finger Tap Test			
Dominant Hand	0.72	0.76	0.67
Non Dominant Hand	0.54	0.51	0.60
Stroop (with outliers)	0.04	0.003	0.36
Stroop (no outliers)	0.49	0.37	0.36
PVSAT	0.03	0.11	0.02
Trail Making A	0.19	0.04	0.29
Trail Making B	0.001	0.22	0.05
Spatial Memory	0.00	0.00	0.30

**Table 3:** Results for linear fit and inter-test interpolated labels.

Activity	Data (250 Hz)	Linear Fit MAE_200_	Linear Fit RAE_200_	Inter-Test Interpolation MAE_200_	Inter-Test Interpolation RAE_200_
Run Up	1,019,002	0.145	0.225	0.134	0.240
Run	732,501	0.151	0.238	0.156	0.232
Run Down	1,843,749	0.130	0.289	0.133	0.167
Walk Up	1,534,500	0.136	0.303	0.125	0.411
Walk	299,997	0.238	0.683	0.235	0.726
Walk Down	20,000	0.219	-	0.239	-
Open Gate	56,750	0.195	0.338	0.199	0.316
Climb Gate	65,249	0.327	0.422	0.313	0.389

## References

[1] Phillips R.O. (2015). A review of definitions of fatigue—And a step towards a whole definition. Transp. Res. Part F Traffic Psychol. Behav..

[2] Caldwell J.A., Caldwell J.L., Brown D.L., Smith J.K. (2004). The Effects of 37 Hours of Continuous Wakefulness On the Physiological Arousal, Cognitive Performance, Self-Reported Mood, and Simulator Flight Performance of F-117A Pilots. Mil. Psychol..

[3] Thomas L.C., Gast C., Grube R., Craig K. (2015). Fatigue Detection in Commercial Flight Operations: Results Using Physiological Measures. Procedia Manuf..

[4] Vural E., Çetin M., Erçil A. (2007). Machine Learning Systems for Detecting Driver Drowsiness. Digital Signal Processing for In-Vehicle Systems and Safety.

[5] Desai A.V., Haque M.A. (2006). Vigilance monitoring for operator safety: A simulation study on highway driving. J. Saf. Res..

[6] Correa A.G., Orosco L., Laciar E. (2014). Automatic detection of drowsiness in EEG records based on multimodal analysis. Med. Eng. Phys..

[7] Von Jan T., Karnahl T., Seifert K., Hilgenstock J., Zobel R. Don’t sleep and drive—VW’s fatigue detection technology. Proceedings of the 19th International Conference on Enhanced Safety of Vehicles.

[8] Chen L.L., Zhao Y., Zhang J., Zou J.Z. (2015). Automatic detection of alertness/drowsiness from physiological signals using wavelet-based nonlinear features and machine learning. Expert Syst. Appl..

[9] Duncan M.J., Fowler N., George O., Joyce S., Hankey J. (2015). Mental Fatigue Negatively Influences Manual Dexterity and Anticipation Timing but not Repeated High-intensity Exercise Performance in Trained Adults. Res. Sports Med..

[10] Park K., Rosengren K.S., Horn G.P., Smith D.L., Hsiao-Wecksler E.T. (2011). Assessing gait changes in firefighters due to fatigue and protective clothing. Saf. Sci..

[11] Smith B.P., Browne M., Armstrong T.A., Ferguson S.A. (2016). The accuracy of subjective measures for assessing fatigue related decrements in multi-stressor environments. Saf. Sci..

[12] Dawson D., Reid K. (1997). Fatigue alcohol and performance impairment. Nature.

[13] Davis M.P., Walsh D. (2010). Mechanisms of Fatigue. J. Support Oncol..

[14] Noakes T.D. (2012). Fatigue is a brain-derived emotion that regulates the exercise behavior to ensure the protection of whole body homeostasis. Front. Physiol..

[15] Hampson D.B., Gibson A.S., Lambert M.I., Noakes T.D. (2001). The Influence of Sensory Cues on the Perception of Exertion During Exercise and Central Regulation of Exercise Performance. Sports Med..

[16] Siirtola P., Laurinen P., Haapalainen E., Röning J., Kinnunen H. Clustering-based activity classification with a wrist-worn accelerometer using basic features. Proceedings of the 2009 IEEE Symp. Comput. Intell. Data Mining, CIDM 2009-Proc..

[17] Noakes T.D. (2000). Physiological models to understand exercise fatigue and the adaptations that predict or enhance athletic performance. Med. Sci. Sports.

[18] Van Cutsem J., Marcora S., De Pauw K., Bailey S., Meeusen R., Roelands B. (2017). The Effects of Mental Fatigue on Physical Performance: A Systematic Review. Sports Med..

[19] Hill A.V., Long C.N.H., Lupton H. (1924). Muscular Exercise Lactic Acid and the Supply and Utilisation of Oxygen. Proc. R. Soc..

[20] Borg G., Borg E. To determine the magnitude of pain with Borg. Proceedings of the Fechner Day 2014—30th Annual Meeting International Society for Psychophysics.

[21] Millet G.Y., Tomazin K., Verges S., Vincent C., Bonnefoy R., Boisson R.C., Gergelé L., Féasson L., Martin V. (2011). Neuromuscular consequences of an extreme mountain ultra-marathon. PLoS ONE.

[22] Venhorst A., Micklewright D., Noakes T.D. (2018). Perceived Fatigability: Utility of a Three-Dimensional Dynamical Systems Framework to Better Understand the Psychophysiological Regulation of Goal-Directed Exercise Behaviour. Sports Med..

[23] Boksem M.A., Tops M. (2008). Mental fatigue: Costs and benefits. Brain Res. Rev..

[24] Möckel T., Beste C., Wascher E. (2015). The Effects of Time on Task in Response Selection—An ERP Study of Mental Fatigue. Nat. Publ. Gr..

[25] Aoyagi M.W., Portenga S.T. (2010). The Role of Positive Ethics and Virtues in the Context of Sport and Performance Psychology Service Delivery. Prof. Psychol. Res. Pract..

[26] Portenga S.T., Aoyagi M.W., Cohen A.B. (2017). Helping to build a profession: A working definition of sport and performance psychology. J. Sport Psychol. Action.

[27] Lambert E.V., Gibson A.S.C., Noakes T.D. (2004). Complex systems model of fatigue: Integrative homoeostatic control of peripheral physiological systems during exercise in humans. Br. J. Sports Med..

[28] Pimenta A., Carneiro D., Neves J., Novais P. (2016). A neural network to classify fatigue from human-computer interaction. Neurocomputing.

[29] Abdulin E. User Fatigue Detection via Eye Movement Behavior. In Proceedings of 33rd Annual ACM Conference Extended Abstracts on Human Factors in Computing Systems.

[30] Gonzalez K., Sasangohar F., Mehta R., Lawley M., Erraguntla M. Measuring Fatigue through Heart Rate Variability and Activity Recognition: A Scoping Literature Review of Machine Learning Techniques. Proceedings of the Human Factors and Ergonomics Society Annual Meeting.

[31] Patel A.N., Howard M.D., Roach S.M., Jones A.P., Bryant N.B., Robinson C.S.H., Clark V.P., Pilly P.K. (2018). Mental State Assessment and Validation Using Personalized Physiological Biometrics. Front. Hum. Neurosci..

[32] Azim T., Jaffar M.A., Mirza A.M. (2014). Fully automated real time fatigue detection of drivers through Fuzzy Expert Systems. Appl. Soft Comput. J..

[33] Enoka R., Duchateau J. (2016). Translating fatigue to human performance. Meical Sci. Sports Exerc..

[34] Zhu Y., Jankay R.R., Pieratt L.C., Mehta R.K. Wearable sensors and their metrics for measuring comprehensive occupational fatigue: A scoping review. Proceedings of the Human Factors and Ergonomics Society Annual Meeting.

[35] Qi W., Su H., Aliverti A. (2020). A Smartphone-Based Adaptive Recognition and Real-Time Monitoring System for Human Activities. IEEE Trans. Hum. Mach. Syst..

[36] Qi W., Su H., Yang C., Ferrigno G., De Momi E., Aliverti A. (2019). A fast and robust deep convolutional neural networks for complex human activity recognition using smartphone. Sensors.

[37] Granacher U., Wolf I., Wehrle A., Bridenbaugh S., Kressig R.W. (2010). Effects of muscle fatigue on gait characteristics under single and dual-task conditions in young and older adults. J. Neuroeng. Rehabil..

[38] Fuller J.T., Bellenger C.R., Thewlis D., Arnold J., Thomson R.L., Tsiros M.D., Robertson E.Y., Buckley J.D. (2017). Tracking performance changes with running-stride variability when athletes are functionally overreached. Int. J. Sports Physiol. Perform..

[39] Heredia-Jimenez J., Latorre-Roman P., Santos-Campos M., Orantes-Gonzalez E., Soto-Hermoso V.M. (2016). Spatio-temporal gait disorder and gait fatigue index in a six-minute walk test in women with fibromyalgia. Clin. Biomech..

[40] Marcora S.M., Staiano W., Manning V. (2009). Mental fatigue impairs physical performance in humans. J. Appl. Physiol. Publ..

[41] Roelands B., De Koning J., Foster C., Hettinga F., Meeusen R. (2013). Neurophysiological determinants of theoretical concepts and mechanisms involved in pacing. Sports Med..

[42] Borghini G., Astolfi L., Vecchiato G., Mattia D., Babiloni F. (2014). Measuring neurophysiological signals in aircraft pilots and car drivers for the assessment of mental workload, fatigue and drowsiness. Neurosci. Biobehav. Rev..

[43] Russell B., McDaid A., Toscano W., Hume P. (2021). Moving the Lab into the Mountains: A Pilot Study of Human Activity Recognition in Unstructured Environments. Sensors.

[44] Wang G., Li Q., Wang L., Wang W., Wu M., Liu T. (2018). Impact of sliding window length in indoor human motion modes and pose pattern recognition based on smartphone sensors. Sensors.

[45] Yoon H., Hwan S., Ho S., Choi J., Jin Y. (2018). Wakefulness evaluation during sleep for healthy subjects and OSA patients using a patch-type device. Comput. Methods Progr. Biomed..

[46] Gordienko Y., Stirenko S., Kochura Y., Alienin O., Novotarskiy M., Gordienko N. (2017). Deep Learning for Fatigue Estimation on the Basis of Multimodal Human-Machine Interactions. arXiv.

[47] van der Westhuizen J., Lasenby J. (2016). A Review of Machine Learning Applied to Time Series.

[48] Ignatov A. (2018). Real-time human activity recognition from accelerometer data using Convolutional Neural Networks. Appl. Soft Comput. J..

[49] Tripathi S., Acharya S., Sharma R.D., Mittal S., Bhattacharya S. Using Deep and Convolutional Neural Networks for Accurate Emotion Classification on DEAP Dataset. Proceedings of the Twenty-Ninth IAAI Conference.

[50] Laurent F., Valderrama M., Besserve M., Guillard M., Lachaux J.P., Martinerie J., Florence G. (2013). Multimodal information improves the rapid detection of mental fatigue. Biomed. Signal Process. Control.

[51] Grobe S., Kakar R.S., Smith M.L., Mehta R., Baghurst T., Boolani A. (2017). Impact of cognitive fatigue on gait and sway among older adults: A literature review. Prev. Med. Rep..

[52] Vanhatalo A., Jones A.M., Burnley M. (2011). Application of Critical Power in Sport What Is the Critical Power Concept. Int. J. Sports Physiol. Perform..

[53] Miyake S., Yamada S., Shoji T., Takae Y., Kuge N., Yamamura T. (2009). Physiological responses to workload change. A test/retest examination. Appl. Ergon..

[54] Gibson A.S.C., Goedecke J.H., Harley Y.X., Myers L.J., Lambert M.I., Noakes T.D., Lambert E.V. (2005). Metabolic setpoint control mechanisms in different physiological systems at rest and during exercise. J. Theor. Biol..

[55] Wickens C.D., Keller J.W., Shaw C. (2015). Human Factors in High-Altitude Mountaineering. J. Hum. Perform. Extrem. Environ..

[56] Apple Research Kit. https://developer.apple.com/researchkit/.

[57] Cherry B.J., Zettel-Watson L., Chang J.C., Shimizu R., Rutledge D.N., Jones C.J. (2012). Positive associations between physical and cognitive performance measures in fibromyalgia. Arch. Phys. Med. Rehabil..

[58] Lee C.Y., Kang S.J., Hong S.K., Ma H., Lee U., Kim Y.J. (2016). A validation study of a smartphone-based finger tapping application for quantitative assessment of bradykinesia in Parkinson’s disease. PLoS ONE.

[59] Leyla A., Kiziltan E. (2016). Polyphasic Temporal Behavior of Finger-Tapping Performance. J. Mot. Behav..

[60] Pageaux B., Marcora S.M., Rozand V., Lepers R. (2015). Mental fatigue induced by prolonged self-regulation does not exacerbate central fatigue during subsequent whole-body endurance exercise. Front. Hum. Neurosci..

[61] Egner T., Hirsch J. (2005). The neural correlates and functional integration of cognitive control in a Stroop task. Neuroimage.

[62] Iancheva D., Trenova A.G., Terziyski K., Kandilarova S., Mantarova S. (2018). Translational validity of PASAT and the effect of fatigue and mood in patients with relapsing remitting MS: A functional MRI study. J. Eval. Clin. Pract..

[63] Gonzales J.U., James C.R., Yang H.S., Jensen D., Atkins L., Thompson B.J., Al-Khalil K., O’Boyle M. (2016). Different cognitive functions discriminate gait performance in younger and older women: A pilot study. Gait Posture.

[64] Corsi P.M. (1972). Memory and the Medial Temporal Region of the Brain. Ph.D. Thesis.

[65] Brunetti R., Del Gatto C., Delogu F. (2014). eCorsi: Implementation and testing of the Corsi block-tapping task for digital tablets. Front. Psychol..

[66] Watkins C.M., Barillas S.R., Wong M.A., Archer D.C., Dobbs I.J., Lockie R.G., Coburn J.W., Tran T.T., Brown L.E. (2017). Determination of vertical jump as a measure of neuromuscular readiness and fatigue. J. Strength Cond. Res..

[67] Kovářová L., Pánek D., Kovář K., Hlinčík Z. (2015). Relationship between subjectively perceived exertion and objective loading in trained athletes and non-athletes. J. Phys. Educ. Sport.

[68] Johnstone J.A., Ford P.A., Hughes G., Watson T., Garrett A.T. (2012). Bioharness^TM^ multivariable monitoring device. Part I: Validity. J. Sports Sci. Med..

[69] Johnstone J.A., Ford P.A., Hughes G., Watson T., Garrett A.T. (2012). Bioharness^TM^ multivariable monitoring device. Part II: Reliability. J. Sports Sci. Med..

[70] Winter D.A. (1984). Kinematic and kinetic patterns in human gait: Variability and compensating effects. Hum. Mov. Sci..

